# In Vitro Evaluation of Sub-Lethal Concentrations of Plant-Derived Antifungal Compounds on FUSARIA Growth and Mycotoxin Production

**DOI:** 10.3390/molecules22081271

**Published:** 2017-07-29

**Authors:** Caterina Morcia, Giorgio Tumino, Roberta Ghizzoni, Assetou Bara, Nesrine Salhi, Valeria Terzi

**Affiliations:** 1 Research Centre for Genomics and Bioinformatics (CREA-GB), Council for Agricultural Research and Economics, Via San Protaso 302, 29017 Fiorenzuola d’Arda (PC), Italy; caterina.morcia@crea.gov.it (C.M.); giorgiotumino@hotmail.it (G.T.); roberta.ghizzoni@crea.gov.it (R.G.); assetou.bara90@gmail.com (A.B.); 2 Faculté des Sciences de la Nature et de la Vie, Université Kasdi Merbah Ouargla, Ouargla 30 000, Algérie; nesrinemed@yahoo.fr

**Keywords:** natural products, *Fusarium graminearum*, *Fusarium sporotrichioides*, *Fusarium langsethiae*, T-2, HT-2 toxins, deoxynivalenol (DON)

## Abstract

Phytopathogenic fungi can lead to significant cereal yield losses, also producing mycotoxins dangerous for human and animal health. The fungal control based on the use of synthetic fungicides can be complemented by "green" methods for crop protection, based on the use of natural products. In this frame, the antifungal activities of bergamot and lemon essential oils and of five natural compounds recurrent in essential oils (citronellal, citral, cinnamaldehyde, cuminaldehyde and limonene) have been evaluated against three species of mycotoxigenic fungi (*Fusarium sporotrichioides*, *F. graminearum* and *F. langsethiae*) responsible for Fusarium Head Blight in small-grain cereals. The natural products concentrations effective for reducing or inhibiting the in vitro fungal growth were determined for each fungal species and the following scale of potency was found: cinnamaldehyde > cuminaldehyde > citral > citronellal > bergamot oil > limonene > lemon oil. Moreover, the in vitro mycotoxin productions of the three Fusaria strains exposed to sub-lethal concentrations of the seven products was evaluated. The three fungal species showed variability in response to the treatments, both in terms of inhibition of mycelial growth and in terms of modulation of mycotoxin production that can be enhanced by sub-lethal concentrations of some natural products. This last finding must be taken into account in the frame of an open field application of some plant-derived fungicides.

## 1. Introduction

Cereals are the most important food source worldwide, with a global 2016 production of 2600 million tonnes [1]. Cereal productions can be negatively affected by several diseases, sustained by fungi, bacteria and viruses. Among fungi that infect cereals in field, Fusaria are successful pathogens, characterized by considerable morphological and physiological variability, and capable of occupying many ecological niches in very different cereal cultivation environments [2,3]. In small-grain cereals, several Fusaria species are etiologic agents of Fusarium Head Blight (FHB), a complex disease resulting in relevant yield losses and in the contamination of the grains with mycotoxins, fungal secondary metabolites dangerous for human and animal health [4]. In particular, the three fungal species *F. sporotrichioides, F. graminearum* and *F. langsethiae* are involved in FHB in a wide range of cultivation environments, including Mediterranean ones, and are responsible for the production of A and B trichothecenes [5]. These sesquiterpenoid molecules contain a tetracyclic 12,13-epoxy ring responsible for the toxicological properties: group A includes T-2, HT-2 toxins, whereas group B includes deoxynivalenol (DON). Type-B trichothecenes seem to be prevalent in several cereal cultivation areas, but type-A trichothecenes have higher toxicity than those of the type-B group [6]. In particular, T-2 and HT-2 toxins are the most toxic among trichothecenes and therefore deserve special attention.

The control and the reduction of such contaminations are high priorities for the cereal production chain. Several different strategies can be adopted to mitigate the problem, including breeding resistant varieties, fungal populations monitoring, adoption of suitable agronomic practices and protocols for crop protection [7]. With reference to this latter point, demethylation inhibitors are among the synthetic fungicides effective in controlling FHB: molecules such as tebuconazole, protoconazole and metaconazole are widely used, alone or in combination [8]. Limitations of these molecules are their high cost, the fact that their effectiveness is strictly dependent on the timely application and on the evolution of resistant fungal populations. This latter possibility suggests that, to overcome multidrug resistance evolution, the range of synthetic fungicides should be widened and complemented with natural fungicides produced by the secondary metabolism of different plant species, such as essential oils and their components. Essential oils, in nature, play an important role in plant-pathogen interaction and in plant protection thanks to their antibacterial, antiviral, antifungal and insecticidal action. It has long been known that many plant essential oils have microbicidal properties [9], which has suggested their use in traditional medicines and in pharmacology, as reviewed by Sharifi-Rad et al. [10]. Several studies have been focused on evaluating the use of essential oils in food conservation [11] and, to a lesser extent, in crop protection [12]. Some of these natural compounds are now principal constituents of antifungal formulations, commercially available and used to inhibit storage fungi and to control various plant diseases [13,14]. Moreover, plant essential oils have been demonstrated to be a useful tool for the control of mycotoxigenic moulds, as reviewed by Prakash et al. [15]. 

In our study, we have considered seven natural products (bergamot and lemon essential oils, citronellal, citral, cinnamaldehyde, cuminaldehyde and limonene) that are recurrent in medicinal and aromatic plants and were selected taking into account their commercial availability, the fact that they are considered as GRAS (Generally Recognized As Safe) and their low costs. 

Bergamot essential oil is extracted from pericarp and mesocarp of *Citrus aurantium* ssp. *bergamia* (Risso & Poiteau) fresh fruit and has limonene, linalyl acetate, linalool, γ-terpinene, and β-pinene as major components [16,17]. Its antimicrobial action has been demonstrated against a wide range of pathogens, including some plant pathogen fungi, such as *Fusarium solani*, *F. sporotrichioides* and *F. oxysporum*, and against food spoilage fungi belonging to the *Aspergillus* genus [18]. 

Lemon essential oil is derived from *Citrus limon* L. and is characterized by limonene, γ-terpinene, β-pinene, α-pinene, citral and myrcene as major components. Antifungal properties of lemon oil have been demonstrated against several phytopatogens, such as *Aspergillus*, *Penicillium*, *Fusarium* spp., *Rhizopus* spp. and *Botrytis* (reviewed by Jing et al.) [19].

Bergamot and lemon essential oils are mixtures of several components that exert antimicrobial action through complex and not yet completely understood molecular mechanisms, resulting in cell permeability alteration, cytoplasmic membranes damage, microbial cell death (reviewed by Calo et al.) [20].

Citronellal is an aldehyde and is the major component of *Cymbopogon*, lemon-scented gum (*Eucalyptus citriodora*), and lemon-scented teatree (*Leptospermum petersonii*) essential oils. Citronellal has been demonstrated effective for the control of rice pathogens, such as *Rhizoctonia* and *Helminthosporium oryzae* [21] and its antifungal activity results in plasma membrane damage, as demonstrated in *Penicillium digitatum* by Wang et al. [22].

Citral is a mixture of the monoterpenic aldehydes geranial and neral and is present in the essential oil of several plants, among others lemon myrtle, *Litsea citrata*, *Litsea cubeba*, lemongrass, lemon tea-tree, *Ocimum gratissimum*, *Lindera citriodora*, *Calypranthes parriculata*. Antifungal properties of citral have been demonstrated in six plant pathogenic fungi (*Magnaporthe grisea, Gibberella zeae, Fusarium oxysporum, Valsa mali, Botrytis cinerea* and *Rhizoctonia solani*) by Li et al. [23]. These researchers found that citral negatively affects the mycelia reducing sugar, soluble protein, chitinase activity, pyruvate content and MDA content of *Magnaporthe grisea*, even if the exact molecular mechanism of action still remains to be determined. 

Cinnamaldehyde occurs in the essential oil present in bark of species belonging to the *Cinnamomum* genus and is currently widely used in cosmetic, food and pharmaceutical industries. Cinnamaldehyde interferes with enzymatic reactions of fungal cell wall synthesis, affecting the morphogenesis and growth in *F. verticillioides*, as demonstrated by Xing et al. [24]. In *Aspergillus flavus*, cinnamaldehyde inhibits radial growth, spore production, mycelium formation, and aflatoxin biosynthesis in a dose-dependent manner [25].

Cuminaldehyde is present in the essential oils of *Cumin cyminum, Eucalyptus*, *Cinnamomum cassia* and others medicinal plants. Marei et al. [26] demonstrated its strong antifungal property against the four plant pathogenic fungi *Rhizoctonia solani*, *Fusarium oxysporum*, *Penicillium digitatum* and *Aspergillus niger.*

These same fungi are inhibited even by limonene, which inhibits fungal enzymes such as pectin methyl esterase, cellulase and polyphenol oxidase, leading to cytoplasm granulation and cytoplasmic membrane damage [26]. Even spoilage mycotoxigenic moulds, such as *Aspergillus flavus* and *A. parasiticus*, are inhibited by limonene treatment [27]. Limonene is a monoterpene cyclic hydrocarbon present in the essential oils of several aromatic and medicinal plants, e.g., *Citrus, Juniperus, Pinus,* but is mainly produced as side product from the citrus juice industry. 

The antifungal properties of several natural compounds are known; however, very limited knowledge is available on the effect of their sub-lethal concentrations on fungal populations. This aspect is of relevance in pathogen control strategies: in the case of demethylation-inhibiting fungicides, a major consequence of their sub-optimal applications has been identified in the development of quantitative resistance [28]. In the case of mycotoxigenic fungi, there are evidences that sub-optimal amounts of azole fungicides can increase mycotoxin production [29,30,31].

The aim of this work was to investigate the influence of seven natural products, including both essential oils and components of essential oils, on the three mycotoxigenic species *F. sporotrichioides, F. graminearum* and *F. langsethiae*. The biological activity of the seven compounds was evaluated measuring their impact on in vitro fungal mycelium growth and on mycotoxin production. In particular, the impact of sub-lethal concentrations of the natural products on the in vitro mycotoxin production was studied.

## 2. Results and Discussion 

Bergamot oil, lemon oil, citronellal, citral, cinnamaldehyde, cuminaldehyde and limonene are all inhibitor of Fusaria growth in vitro, as reported in Table 1. 

The three fungal species showed different levels of sensitivity to the different treatments. The data obtained indicated that, among the different compounds, there is variability in the concentrations effective to reduce the mycelial growth of the three fungi. 

**Figure 1:**
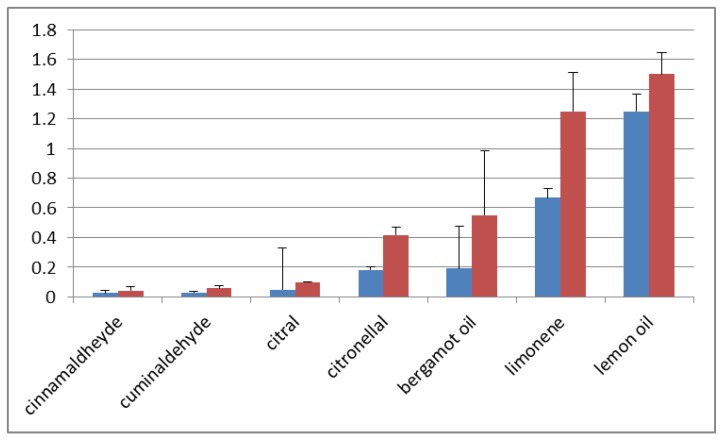
Inhibitory concentrations of the natural products on Fusaria mycelial growth. The mean concentrations (expressed as mL of compounds/100mL medium) that give a 50% inhibition (blue bars) and a 100% inhibition (red bars) of the mycelium growth in vitro are shown. The values are calculated as means of the three Fusaria species.

Figure 1 reports the mean concentrations able to reduce 50% and 100% fungal growth, calculated starting from the values obtained in all the fungal strains. The different natural products have different potency and the following scale can be indicated:

cinnamaldehyde > cuminaldehyde > citral > citronellal > bergamot oil > limonene > lemon oil

The group of aldehydes has therefore stronger antifungal activity in comparison with the hydrocarbon limonene and with the bergamot and lemon essential oils, in which hydrocarbons are the major components.

This finding is in agreement with the scale of potency indicated by Kurita and Koike [32], further confirmed by Morcia et al. [33]:

phenols > alcohols > aldehydes > ketones > ethers > hydrocarbons.

According to our study, aldehydes are confirmed to be antifungal molecules characterized by higher level of potency in comparison with hydrocarbons. Lu et al. [34] agree that essential oils containing phenolic and aldehyde components (thymol, cinnamaldehyde, cuminaldehyde and citral-a) have higher level of antimicrobial activity against *P. parasitica* var. *nicotianae* than other essential oils containing alcohol and terpene compounds. Lee et al. [35], studing the antifungal activity of *Myrtaceae* essential oils, found that aldehyde and alcohol compounds are more toxic for *Phytophtora infestans*, *Cryponectria parasitica* and *Fusarium circinatum* than hydrocarbons. In agreement with our results, these authors observed that, among the aldehyde compounds evaluated, citral is more active than citronellal. 

Our results confirm that the seven compounds tested are effective in contrasting Fusaria growth in vitro. A number of works report that several plant essential oils, at proper concentrations, inhibit both the fungal growth and the mycotoxin production, as reviewed by Prakash et al. [14]. However, the impact of sub-lethal concentrations on mycotoxin synthesis has not been so deeply studied. Therefore, we have quantified the in vitro mycotoxin productions of the three Fusaria strains when the fungi are growing in presence of sub-lethal concentrations of the seven products. 

**Figure 2:**
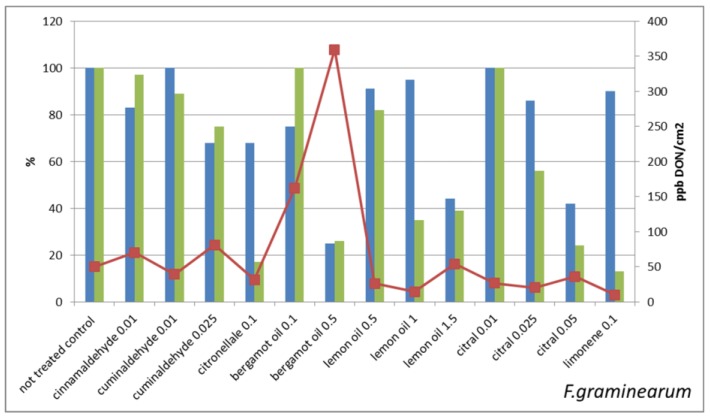
Impact of sub-lethal product concentrations on *F. graminearum* growth and mycotoxin production. Blue bars indicate the mean values of mycelium in vitro growth (expressed as percentage of growth respect to untreated control) of *Fusarium graminearum* treated with sub-lethal concentrations of the indicated natural products. Green bars represent the mycotoxin production (expressed as percentage of deoxynivalenol (DON) respect to untreated control) and the line chart indicates the DON concentration (expressed in ppb) per mycelium unit area (expressed in cm^2^).

**Figure 3:**
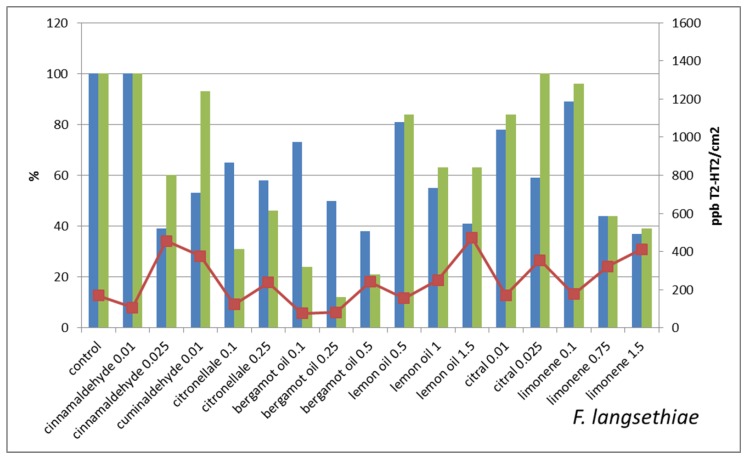
Impact of sub-lethal product concentrations on *F. langsethiae* growth and mycotoxin production. Blue bars indicate the mean values of mycelium in vitro growth (expressed as percentage of growth respect to untreated control) of *Fusarium langsethiae* treated with sub-lethal concentrations of the indicated natural products. Green bars represent the mycotoxin production (expressed as percentage of T-2, HT-2 respect to untreated control) and the line chart indicates the T-2, HT-2 concentration (expressed in ppb) per mycelium unit area (expressed in cm^2^).

Figure 2 reports the mycelium growth of *Fusarium graminearum* (expressed as percentage of growth respect to control) and the DON production (expressed both as percentage of total DON production respect to control and as ppb of DON per mycelium unit area (cm^2^)). The correlation between the reduction of mycelium growth and of total mycotoxin production (r = 0.53) is significant at 0.05 level. In *Fusarium langsethiae* (Figure 3) the correlation between reduction of mycelium growth and of T-2,HT-2 production is significant at 0.05 level (r = 0.59). 

**Figure 4:**
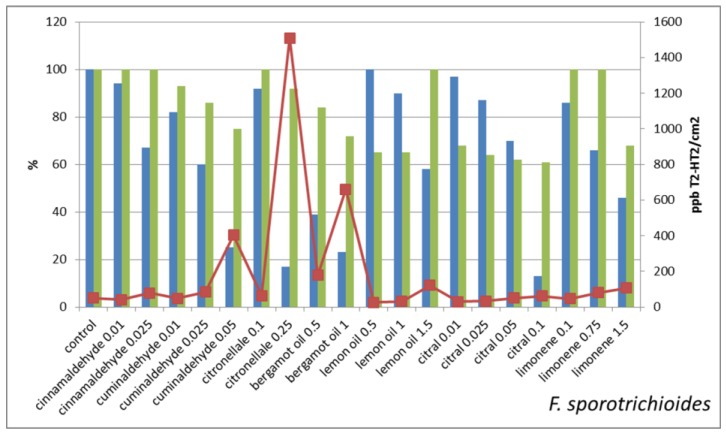
Impact of sub-lethal product concentrations on *F. sporotrichioides* growth and mycotoxin production. Blue bars indicate the mean values of mycelium in vitro growth (expressed as percentage of growth respect to untreated control) of *Fusarium sporotrichioides* treated with sub-lethal concentrations of the indicated natural products. Green bars represent the mycotoxin production (expressed as percentage of T-2, HT-2 respect to untreated control) and the line chart indicates the T-2, HT-2 concentration (expressed in ppb) per mycelium unit area (expressed in cm^2^).

On the contrary, in *Fusarium sporotrichioides* (Figure 4) the correlation between reduction of mycelium growth and of T-2, HT-2 production is not significant at 0.05 level (r = 0.19). 

These results suggest that the concomitant reduction of mycelium growth and of mycotoxin synthesis is not always occurring: some of the sub-lethal treatments seem to trigger mycotoxin synthesis. This is evident when the mycotoxin concentrations per cm^2^ of mycelium are considered. More in details, the Fusaria strains that we have treated with sub-lethal concentrations of antifungal molecules show different behaviors that can be schematized in four classes, i.e.:

1. the mycotoxin concentration/cm^2^ in treated is lower than in untreated control;
2. the mycotoxin concentration/cm^2^ in treated is very close to untreated control;
3. the mycotoxin concentration/cm^2^ is two–three times higher in treated in comparison with untreated control;
4. the mycotoxin concentration/cm^2^ in treated is orders of magnitude higher than in untreated control

The treatments that strongly reduce mycelium growth all belong to this last class, e.g., *F. graminearum* treated with 0.5% bergamot oil and *F. sporotrichioides* treated with 0.25% citronellal and with 1% bergamot oil. On the contrary, treatments that slightly reduce the mycelium growth result in lowering the mycotoxin production in comparison with untreated control, e.g., *F. graminearum* treated with cuminaldehyde 0.01% or with citral 0.01% or limonene 0.1% and *F. sporotrichioides* treated with lemon oil 0.5%. 

The modulation of in vitro mycotoxin synthesis by sub-lethal concentrations of synthetic fungicides has been evaluated in several studies, obtaining contrasting results [36]. For example, inhibition or reduction of 3-ADON and of diacetoxyscirpenol have been observed in *F. graminearum* strains grown in presence of dicloram (500 μg/mL) or vinclozolin (250 μg/mL) [37] and of tebuconazole (1 μg/mL), thiabendazole (1 μg/mL), benomyl (1 μg/mL), or prochloraz (5 μg/mL) [38]. On the contrary, tebuconazole (0.1 μg/mL) and difenoconazole (0.1 μg/mL) increase the production of 3-ADON in cultures of *F. culmorum* [39]. *Fusarium sporotrichioides* in presence of sub-lethal level of tridemorph (30 to 50 μg/mL) [40] or carbendazim (5 μg/mL) [41] increases the T-2 toxin production. Magan et al. [42] reported that sub-optimal levels of fungicides stimulated DON production by *F. culmorum* in wheat grain. Kulik et al. [43] reported that sub-lethal concentrations of azoles induce *tri* genes transcription, resulting in increased level of trichothecenes in *F. graminearum*. Ochiai et al. [29] found that a sub-lethal dose of tebuconazole increases *tri* transcript level in *F. asiaticum*, and Becher et al. [44] observed higher level of NIV production in *F. graminearum* NIV chemotype adapted to sub-lethal concentrations of tebuconazole. 

In general, mycelia growth retardation or inhibition due to essential oils treatments significantly decrease mycotoxin production [45]. However, the fungal growth inhibition and toxins production inhibition do not always occur together. Sumalan et al. [46] found that *Thymus vulgaris, Mentha piperita* and *Cinnamomum zeylanicum* produced the higher mycotoxin inhibition in Fusaria, but the most fungicidal effect was recorded for *Salvia officinalis*. Hope et al. [47] found that sub-optimal cinnamon oil concentration can significatively reduce the growth of *F. culmorum* and *F. graminearum*, but enhance their toxins production. Prakash et al. [15] found that low concentration of *P. betle* essential oil induces overproduction of AFB1 in *A. flavus*. Stimulation of aflatoxigenesis in *Aspergilla* has been observed even by Garcia et al. [48] in presence of *E. arvense* and *S. rebaudiana* extracts.

It is known that adverse environmental conditions, among others oxidative stress and N-starvation stress, can trigger mycotoxin production in fungi [49]. It can therefore be hypothesized that low fungicide doses can be a stress condition which increases the toxin synthesis as a defense mechanism by the fungus [17,24]. Moreover, the independency of the mycelium growth and toxin production cellular processes has been postulated by Ferruz et al. [50]. These authors in fact demonstrated a different modulation of fungal growth and T-2, HT-2 production in *Fusarium langsethiae* and *F. sporotrichioides* treated with different concentrations of phenolic acids. The regulation of toxin synthesis is in fact at transcriptional level and independent from that of fungal growth. 

## 3. Material and Methods

### 3.1. Fungal Strains 

*Fusarium sporotrichioides* (ITEM Collection n. 692), *F. langsethiae* (ITEM Collection n. 11020) and *F. graminearum* (ITEM Collection n. 6477) monosporic reference strains were kindly provided by Dr. Antonio Moretti, ISPA-CNR (Institute of Sciences for Food Production, National Research Council, Bari, Italy) and belong to the toxigenic fungi ISPA ITEM Collection. 

### 3.2. Chemicals

The following compounds were purchased from Sigma-Aldrich S.r.l. (Milan, Italy): (+/−)- citronellal (code 27470); bergamot oil (code W215309); lemon oil (code W262528); citral (code W230316); cinnamaldehyde, (code W228613); cuminaldehyde (code 135178) and (R)–(+)–limonene (code 62118).

### 3.3. Evaluation of the Effect of Compounds on In Vitro Fungal Growth

Colony plugs (0.5 cm diameter) were extracted with a cork borer from the margin of fungal colonies grown on Potato Dextrose Agar (PDA) medium (Liofilchem, Teramo, Italy) for 1 week at 24 °C. The inocula were placed in the center of Petri dishes (one plug per plate) amended with 0.5% Tween 20 and with the products at percentages ranging from 0 to 1.5%. The plates were then sealed with Parafilm and incubated at 24 °C under fluorescent light (12 h photoperiod). The diameters of fungal colonies were measured in two perpendicular directions up to 6 days post-inoculation. All the experiments were conducted twice in triplicate. The effect of different molecules and of different concentrations of compounds was expressed as percentage inhibition, calculated according to the formula: I = [(C − T)/C] × 100, where I is the percentage of inhibition, C is the control plate colony diameter in mm, and T is the treated plate colony diameter in mm. The EC_50_ values were evaluated after probit/logit data linearization [51].

### 3.4. Evaluation of the Effect of Compounds on In Vitro Mycotoxin Production

The whole mycelium, including the underlying PDA, was collected from control and treated plates six days after inoculation with Fusaria. 

T-2,HT-2 content was determined in plates inoculated with *Fusarium sporotrichioides* and *Fusarium langsethiae*, whereas DON content was determined in plates inoculated with *Fusarium graminearum.*

The collected samples were extracted with 5 volumes of 70% methanol for the analysis of T-2,HT-2 and 5 volumes of distilled water for the analysis of DON and vigorously shaken for 15 min before filtering.

The amount of T-2,HT-2 toxins (as sum of toxins) was determined using the kit Veratox® for T-2,HT-2, (Product code 8230, Neogen Corporation, Lansing, Michigan, USA) a competitive direct enzyme-linked immunosorbent assay (ELISA). The photometric reading was done at 630 nm, according to the manufacturer’s instructions.

The amount of DON was determined using the kit RIDASCREEN® DON (Product code R5906, R-Biopharm AG, Darmstadt, Germany), a competitive enzyme immunoassay (ELISA). The photometric reading was done at 450 nm, according to the manufacturer’s instructions.

The photometric readings were done with a ChroMate Model 4300 microplate reader (Awareness Technology INC. Palm City, FL, USA) and the toxin concentrations were calculated using the RIDA® SOFT Win. net software (code Z9996, R-Biopharm AG, Darmstadt, Germany).

## 4. Conclusions

We have demonstrated that cinnamaldehyde, cuminaldehyde, citral, citronellal, bergamot oil, limonene and lemon oil, at proper concentrations, can inhibit the in vitro mycelium growth of three Fusaria species responsible for mycotoxin contamination of cereals. However, at sub-lethal concentrations some of the treatments can increase mycotoxin production. This specific effect, observed only in some of the product–fungus combinations evaluated, deserves deeper study, in view of the practical application of such molecules for crop protection.

## Figures and Tables

**Table 1:** EC_50_ * and EC_100_ ** (expressed as mL of compounds per 100 mL medium) of the seven products on in vitro growth of *F. sporotrichioides, F. graminearum* and *F. langsethiae*.

	*Fusarium sporotrichioides*		*Fusarium graminearum*		*Fusarium langsethiae*	
	EC50	EC100	EC50	EC100	EC50	EC100
cuminaldehyde	0.031	0.075	0.046	0.075	0.015	0.025
cinnamaldehyde	0.036	0.05	0.02	0.025	0.02	0.05
lemon oil	1.5	>1.5	1.3	>1.5	0.95	>1.5
citral	0.067	0.15	0.05	0.1	0.027	0.05
limonene	1	>1.5	0.5	0.75	0.5	>1.5
bergamot oil	0.12	0.25	0.22	0.7	0.23	0.7
citronellal	0.32	0.5	0.12	0.25	0.11	0.5

* Effective Concentration (EC) for 50% fungal growth inhibition; ** Effective Concentration for 100% fungal growth inhibition.
